# Unsaturated C_3,5,7,9_-Monocarboxylic Acids by Aqueous, One-Pot Carbon Fixation: Possible Relevance for the Origin of Life

**DOI:** 10.1038/srep27595

**Published:** 2016-06-10

**Authors:** Christopher Scheidler, Jessica Sobotta, Wolfgang Eisenreich, Günter Wächtershäuser, Claudia Huber

**Affiliations:** 1 Lehrstuhl für Biochemie, Technische Universität München, Lichtenbergstraße 4, D-85747 Garching Germany; 2 2209 Mill Race Drive, Chapel Hill, NC 27514, USA

## Abstract

All scientific approaches to the origin of life share a common problem: a chemical path to lipids as main constituents of extant cellular enclosures. Here we show by isotope-controlled experiments that unsaturated C_3,5,7,9_-monocarboxylic acids form by one-pot reaction of acetylene (C_2_H_2_) and carbon monoxide (CO) in contact with nickel sulfide (NiS) in hot aqueous medium. The primary products are *toto*-olefinic monocarboxylic acids with CO-derived COOH groups undergoing subsequent stepwise hydrogenation with CO as reductant. In the resulting unsaturated monocarboxylic acids the double bonds are mainly centrally located with mainly trans-configuration. The reaction conditions are compatible with an origin of life in volcanic-hydrothermal sub-seafloor flow ducts.

Carbonaceous meteorites have been found to contain saturated, aliphatic C_2–12_-monocarboxylic acids with an excess of branched structures and with abundancies that decrease with increasing carbon atom numbers^1^,^2^,^3^. The relevance of these findings for the origin of life came into its own when hydrophobic residues of chloroform extracts of carbonaceous meteorites were shown to form membranous structures in aqueous media^4^. Meteoritic sources would, however, have been slow at releasing their lipids into the primitive ocean with the result of a highly diluted lipid solution, and possible concentration processes would have been countermanded by massive sedimentation, notably of adsorptive volcanic ashes^5^. A promising alternative emerged with the finding that monocarboxylic acids are formed at 400 °C by contacting metallic iron from certain meteorites with CO and H_2_ (1:1)^6^. The characteristics, however, of an absence of water (except for reaction water) and of a presence of anhydrous (hygroscopic) K_2_CO_3_^6^ speak against hydrothermal vent scenarios for these Fischer-Tropsch reactions. Subsequent reports of a Fischer-Tropsch formation of monocarboxylic acids under water-saturated hydrothermal vent conditions turned out to be problematic because the monocarboxylic acids appear to be formed by gas-solid reactions at catalytic steel surfaces of the reactor wall^7^,^8^. Bearing in mind that the biosynthesis of fatty acids from acetyl-CoA proceeds in C2-increments we probed acetylene as simple, yet highly reactive primordial C2-precursor.

## Results and Discussion

### Synthetic reactions

**Figure 1:**
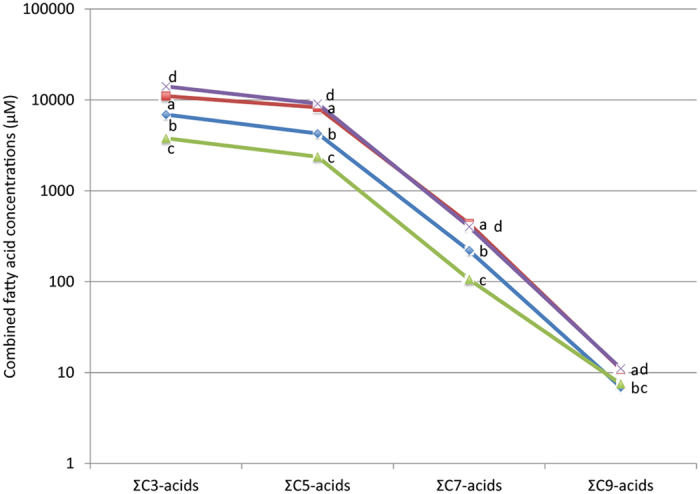
Combined fatty acid concentrations. Total yields of C_3,5,7,9_-monocarboxylic acids of runs a to d are plotted on a logarithmic scale: a: NiS, pH8.8; b: NiS, pH6.7; c: coprecipitated NiS and α-Ni(OH)_2_, pH8.3; d: NiS, precipitated onto β-Ni(OH)_2_, pH8.9; for detailed information see Suppl. Table S1.

We reacted an aqueous suspension of freshly precipitated NiS with C_2_H_2_ and CO as gas phase at a combined gas pressure of ~1 bar at room temperature, followed by heating to 105 °C under an autogenous gas/steam pressure of ~2.5 bar for 7 days. A glass reactor was used in order to avoid artifacts by catalytic reactor walls. The aqueous medium was not buffered and its pH developed autogenously to an end-pH that was measured. After freeze-drying of the supernatant organic products in the residue were silylated and analyzed by gas chromatography-mass spectrometry (GC-MS). The analysis revealed the presence of a suite of C_3,5,7,9_-monocarboxylic acids, their chain length increasing by increments of two C-atoms (see Table 1 and for more detailed information Suppl. Tables S1 and S2, Fig. S4). All detected C_3,5,7,9_-monocarboxylic acids sum up to a concentration of up to about 20 mM, (for detailed conversion rates see Suppl. Table S3). Runs with ^13^CO and D_2_O ascertained that these products are genuine reaction products. In the absence of C_2_H_2_ and/or CO they are not formed. The yields of the monocarboxylic acids show a decrease with increasing chain length (Fig. 1).

### Ancillary investigations

For safety reasons (danger of explosion) and for technical reasons the reactions had to be carried out at low partial pressure (<1 bar) of C_2_H_2_. At a high sub-seafloor pressure (>1000 bar) yields and chain lengths would be increased, because of negative volumes of reaction and activation^9^,^10^. Productivity of the reaction is pH-sensitive with highest yields in the near neutral pH range of about 6.5 to 9, increasing from pH 6.7 (run b) to pH 8.8 (run a). Much stronger alkaline conditions disfavor reaction yields as well as chain elongation. With freshly precipitated α-Ni(OH)_2_ (run e) only C_3,5_-acids were formed, and in lower yields than with freshly precipitated NiS. With coprecipitated NiS and α-Ni(OH)_2_ in a molar ratio of 1:1 (run c) yields were diminished compared to NiS alone. Apparently, α-Ni(OH)_2_ acts as antagonist. With aged β-Ni(OH)_2_ alone (run f) only C_3_-acids were formed, and in lower yields than with α-Ni(OH)_2_. However, with β-Ni(OH)_2_ as carrier for freshly precipitated NiS the yields were slightly increased (run d compared to run a). These comparisons point to a wide range of compositions for the exploration of more effective catalysts. Against the backdrop of previous production of organics by reaction of C_2_H_2_/H_2_O/CO with non-sulfidic Ni-catalysts in organic reaction media^11^,^12^, it is surprising that heterogeneous catalysis by NiS in hot water generates elongated, unsaturated monocarboxlic acids, which undergo *in situ* hydrogenation with the same catalyst and with CO as reductant.

### Proposed mechanism

**Figure 2:**
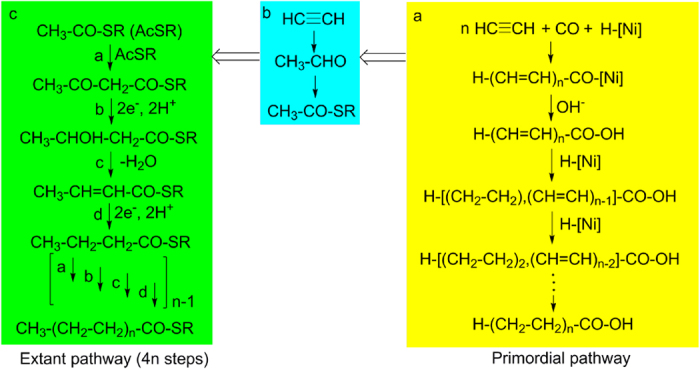
Evolution of monocarboxylic acid biosynthesis. (**a**) Primordial one-pot pathway; (**b**) Conversion of acetylene to acetyl-thioester; (**c**) Extant biosynthesis dependent on acetyl-thioester condensation. [Ni] signifies a catalytic nickel complex (unknown nuclearity, ligand sphere, oxidation state and relationship to mineral surfaces) that converts to H-[Ni] (with hydride ligand) by oxidation of CO to CO_2_; ⇒ signifies an evolutionary transformation.

We suggest an organo-metal reaction mechanism (Fig. 2a), wherein CO acts as carbon source for the carboxyl group and also as reducing agent via hydride transfer. When run a was repeated with the addition of H-(CH=CH)_2_-COOH, but without C_2_H_2_, the same suite of C_5_-monocarboxylic acids was formed with the same quantitative relationships as in run a with C_2_H_2_ (Δ^3^ > Δ^4^ > Δ^2^). Therefore, we suggest *toto*-olefinic H-(CH=CH)_n_-CO-[Ni] as early intermediate, which is hydrolyzed to the corresponding free acid. Subsequently, the free *toto*-olefinic monocarboxylic acids undergo stepwise hydrogenation. The *toto*-olefinic monocarboxylic acids are highly reactive and have a propensity to undergo self-condensation and resinification as established for 2,4,6-heptatrienoic acid and its derivatives^13^,^14^. Therefore, the yield of long-chain monocarboxylic acids is expected to increase with increasing rates of hydrogenation. For n = 1–3 the free *toto*-olefinic mono-carboxylic acids have been detected. A mechanism of electrocyclization and aromatization of *toto*-olefinic monocarboxylic acids with the penultimate double bond in cis-configuration explains the formation of benzoic acid (n = 3)^13^ as well as cinnamic acid and hydrocinnamic acid (n = 4).

The mechanism explains the results with the additional proposition that hydrogenation rates follow three rules. *Rule 1:* The rate is higher if hydrogenation involves the α-carbon atom rather than only more distal carbon atoms. *Rule 2:* Hydrogenation of conjugated double bonds is favored over hydrogenation of isolated double bonds. *Rule 3:* Conjugated double bonds undergo preferably end-to-end hydrogenation. These rules jointly explain why the yields of the mono-unsaturated C_5_-acids (Suppl. Table S1) follow the order Δ^3^ > Δ^4^ > Δ^2^. Moreover, they show that with increasing chain length the proportion of saturated fatty acids decreases sharply and that regioisomers with double bonds in the middle of the carbon chain are favored. Remarkably, double bonds with trans-configuration are also favored. Finally, these rules mean also that multiple double bonds are mainly conjugated.

### Primordial Sources of Starting Materials

We now turn to the possible sources for reactants and catalysts. In the extant atmosphere of the Earth acetylene occurs only in trace amounts close to the detection limit, but much higher levels of atmospheric acetylene have been suggested as contained in a presumptive methane-rich primordial atmosphere due to photolysis of methane^15^. Acetylene has also been detected in extant fumarolic gases^16^, in volcanic glasses^17^, and as products of volcanic simulation experiments^18^. Acetylene has been suggested as product of hydrolysis of calcium carbide (CaC_2_) in the context of the Archaean eon^15^ and in the context of the Hadean eon^19^,^20^. CaC_2_ may be formed by the following transformations in the hot Hadean mantle^21^:


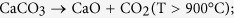



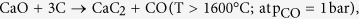


CaC_2_ would have become subsequently emplaced in the Hadean crust to later come in contact with the aqueous vent fluid. The stoichiometrically formed CO would have been continuously removed from the sites of CaC_2_ formation by diffusion and volcanic exhalation. CaC_2_ is a member of the class of acetylenic carbides, i.e. the calcium salt of acetylene, consisting of a lattice of Ca^2+^ cations and C_2_^2−^ anions. It undergoes facile hydrolysis with cold neutral water to form exclusively acetylene^21^. CaC_2_ is distinguished from iron carbides. The latter have been hydrolyzed with concentrated DCl in D_2_O and fully deuterated, saturated C_3–7_-hydrocarbons have been reported as products^22^.

In the presence of graphite the molar ratio of CO:CO_2_ increases with increasing temperature and decreasing pressure. It assumes for example a value of 1:1 at 1200 °C and 2 kbar, or at 900 °C and 0.1 kbar^23^. The Hadean volcanic exhalations containing a high ratio of CO:CO_2_ would eventually have been mixed with relatively cold cycling water in volcanic-hydrothermal flow ducts. The resulting quenching effect would have prevented equilibration of the CO:CO_2_ ratio to low-temperature values^24^.

Nickel is the second-most abundant transition metal (after iron) in the Solar System^25^ and in the crust of the Earth^26^. Iron-nickel sulfides are among the earliest stages of mineral evolution^27^. Therefore, nickel sulfides would have been abundant in the Hadean crust to come in contact with volcanic-hydrothermal vent fluids. Nickel ions have been found to leach out of crustal minerals into hydrothermal vent fluids^28^ and it has been proposed that under the very hot conditions of the Hadean Earth nickel transport in hydrothermal fluids was much more intense than today^29^. Upon contact with H_2_S nanoparticulate nickel sulfide precipitates^30^.

### Evolutionary Considerations

Acetylene utilization has been detected in extant microbial phyla (for review see ref. 15). It shows a characteristic kinetic isotope effect^31^. *Pelobacter acetylenicus* utilizes acetylene as sole carbon and energy source by means of a tungstopterin enzyme that hydrates acetylene to acetaldehyde, which in turn converts to acetic acid plus ethanol. This enzyme still bears the mark of an original redox function^32^. A possibly related relic is provided by the [FeS]-enzyme IspH for the last step in the non-mevalonate pathway to isoprenoid lipids^33^. While it is today clearly a redox enzyme catalyzing the conversion of 1-hydroxy-2-methyl-2(*E*)-butenyl 4-phosphate into a mixture of isopentenyl diphosphate and dimethylallyl diphosphate, it also has an ability to hydrate acetylene^34^. These two enzymes may reflect a deep history of enzyme recruitments and functional recruitments.

Based on the above considerations we project that acetylene utilization may have been a prominent feature of the early metabolism. Hadean volcanic-hydrothermal vent fluids, laden with CO and C_2_H_2_, would have passed through myriads of flow zones. Thereby they would have come in contact with NiS under conditions suitable for the formation of the type of monocarboxylic acids here reported, but perhaps of greater lengths due to high reaction pressure (>1000 bar). These then would have been carried along by the fluid flow in the style of reaction chromatography, the short ones travelling faster than the longer ones^35^.

Let us consider now the possible function of the here reported monocarboxylic acids in the course of the cellularization of life. At the outset we note that the experimentally detected monocarboxylic acids are short (C_3–9_) and that under the chosen experimental conditions of a low partial pressure of acetylene (~0.6 bar at 105 °C) the productivity for the C_9_-monocarboxylic acids is low. It is not unrealistic, however, to expect that future explorations for more effective NiS catalysts may be successful and that higher productivities and greater chain lengths may result at a higher partial pressure of C_2_H_2_ and at a high total pressure (>1000 bar). With these provisos in mind we distinguish here two alternative scenarios for the origin of life.

#### Heterotrophic origin in a prebiotic broth

In this scenario it is assumed that lipid membrane vesicles form *ab initio*. Therefore, the concentration of dissolved monocarboxylic acids in the prebiotic broth must be high enough and their chain length great enough to be able to exceed the critical vesicle concentration (CVC). It has been found that decanoic acid has a CVC at pH 7.2 of ~20 mM^36^, while the CVCs are still higher for nonanoic acid and octanoic acid^37^. The CVC has been shown to be lowered by the inclusion of hydrocarbons^4^, which are known to form by Fischer-Tropsch reactions under hydrothermal conditions^7^ or of the alcohol equivalent of the monocarboxylic acid^37^. Further, the CVC is lowered by the presence of short monocarboxylic acids (e.g. those with 3 to 7 carbon atoms that are reported here) in the solution^36^, which may contribute to reach the saturation level of the disturbance of the structure of liquid water that is required for vesicle formation. With the concentrations of monocarboxylic acids as reported here *ab initio* vesicle formation from solution is questionable.

Aside from the above physico-chemical restriction of heterotrophic *ab initio* vesicle formation from the kind of short monocarboxylic acids here reported, we should note a more principle topological shortcoming of this approach. By the logic of this approach lipid production and supply is located external to the vesicles in question. Growth and reproduction of early vesicles would therefore require lipid nutrients to enter the membrane from the outside with the need for a flipping of lipid molecules from the outer leaflet to the inner leaflet. This outside-in process is the topological opposite of extant cell membrane growth of non-parasitic microbes, which occurs exlusively inside-out, i.e. by the internal synthesis of the lipid molecules followed by their entry into the inner leaflet with subsequent flipping from the inner leaflet to the outer leaflet. This topological difference correlates immediately with a thermodynamic difference. Membrane growth by lipid molecule insertion requires a driving force, which can only be provided by a sufficient lipid concentration. Given the restricted vesicle volume the inside-out process will easily satisfy the concentration requirement, ultimately driven by the synthetic chemical potential of the metabolism. In case of outside-in growth the outside volume is in principle an unrestricted diffusion space with the consequence of unending dilution.

#### Autotrophic origin in a volcanic-hydrothermal fluid flow

In this scenario it is assumed that cellularization is preceded by a surface metabolism with a cascade of intervening functional steps as precursors of the eventual lipid function. The first step in this hypothetical cascade consists of a lipophilization of the catalytic mineral surface by the accumulation of surface-bonded monocarboxylic acids that operate like a two-dimensional hydrophobic solvent^38^. All here reported monocarboxylic acids, even the short ones, are capable of contributing to such surface lipophilization. As a consequence the activity of H_2_O (and of H_3_O^+^ or OH^−^) is lower at the mineral-water interface than in bulk water, thereby protecting hydrolytically sensitive constituents, notably organo-metal intermediates and condensation products. By this collectively autocatalytic effect ever-longer monocarboxylic acids would form in ever-greater proportions. Eventually, a state would be reached, wherein monocarboxylic acids with lipid function form surface-bonded bilayer membranes^38^. At this stage the above-mentioned saturation effects^36^ may come into play. Eventually, semi-cellular structures would form, with a cytosol bounded partly by a lipid membrane and partly by a mineral surface. Still later, evolutionary precursors of true cellular entities with internal catalytic NiS would emerge^38^. Throughout this evolutionary cascade the principle of continuity (topological, structural, nutritional, catalytic and biochemical) would be maintained. Monocarboxylic acids would form first on the open NiS surfaces, later on the NiS surfaces inside the semi-cellular structures, still later on NiS particles inside membrane vesicles, and finally by intracellular enzyme catalysis. Throughout this cascade membrane growth would have proceeded as today — inside-out.

With the cooling of the Earth the acetylene nutrient would have vanished in most habitats of life and the biosynthesis of lipids by organo-metal C2-incremental acetylene fixation would have been replaced by enzymatic C2-incremental acetyl-CoA condensation (Fig. 2c). This gradual transformation would have been mediated by hydration of acetylene to acetaldehyde, followed by oxidative reaction with a mercaptan to form an acetyl-thioester (Fig. 2b). Moreover, we note that extant membranes of the domains Bacteria and Archaea typically comprise a mixture of fatty acid lipids and that a mixture of an even-numbered monocarboxylic acid and an uneven-numbered monocarboxylic acid shows a lowered CVC compared to the single even-numbered case^36^. Therefore, a gradual transition from uneven-numbered to even-numbered carbon chains would not have violated the principle of evolutionary continuity.

Monocarboxylic acids previously invoked in the context of the cellularization of life have been exclusively saturated. By contrast, the here reported synthetic pathways begin by the formation of *toto*-olefinic monocarboxylic acids that undergo subsequent incremental hydrogenation of their double bonds. With the C_3,5_-monocarboxylic acids the fully hydrogenated, saturated state was reached. But with the higher C_7–9_-monocarboxylic acids the saturated states are not reached and the products consist of mixtures of unsaturated monocarboxylic acids with one, two or three double bonds. The double bonds have mainly trans-configuration and multiple double bonds are mainly conjugated. These insights open hitherto unexplored avenues of research into cellularization.

Extant cell membranes of the domains Bacteria and Eukarya are characterized by the presence of natural unsaturated fatty-acyl lipids within membranes of saturated lipids. The unsaturated monocarboxylic acids reported here agree with natural unsaturated lipids with regard to the location of the double bonds in the middle of the hydrophobic tail. While in the here reported synthetic pathway the unsaturated C_>3_-monocarboxylic acids are the exclusive reaction products, the natural unsaturated fatty acids are produced in anaerobic bacteria as minor components by special variants of the fatty acid synthesis machinery.

The natural unsaturated fatty acids have mainly cis-configuration that causes a kinked structure with the effect of a decrease of the membrane packing density. This means an increase of membrane fluidity, as it is required by a mesophilic lifestyle. The trans-unsaturated monocarboxylic acids are actually more similar to their saturated monocarboxylic acid counterparts in terms of their molecular structure and in terms of their effect on membrane properties^39^. With the presence of multiple trans-unsaturations, in their carbon chain the molecules become overall more rigid and less bulky. Now, when the multiple trans-unsaturations are conjugated, as projected for the type of reactions here reported, the carbon chains are even more rigid and rather flat with the result of greater membrane compactness. This provides an outlook to primordial membranes of monocarboxylic acids with conjugated polyunsaturations that may well have properties that are more in tune with the requirements of a (hyper)thermophilic lifestyle. These considerations are speculative, but suitable for empirical verification or falsification.

The chemical reaction here reported is by itself not restricted to any particular scenario as long as the required materials and conditions are present. When viewed, however, in the Iron-Sulfur World context of a volcanic-hydrothermal flow scenario for a chemo-autotrophic origin of life^24^,^35^,^38^,^40^, the here presented reactions show a considerable coherence with previously reported synthetic reactions (nutrients in volcanic-hydrothermal fluid flows and catalytic minerals from crustal flow ducts): Lower alkyl mercaptans form from CO_2_ and H_2_S with FeS^41^; NH_3_ forms from N_2_ with FeS/H_2_S^42^; CH_3_-CO-SCH_3_ forms from CO/CH_3_SH with (Fe,Ni)S^43^; amino acids form from HCN with NiS^44^; amino acids are activated by CO/H_2_S/NiS to form peptides that engage in a peptide cycle by undergoing N-terminal degradation by CO/H_2_S/NiS^45^. This degree coherence may be viewed as a road sign to the origin of life in an open flow system: chemically singular and chemically predetermined.

## Methods

In a typical run a 125 ml glass serum bottle was charged with 0.5 or 1.0 mmol NiSO_4_ • 6H_2_O and closed with a silicon stopper. Additionally or alternatively, β-Ni(OH)_2_ was charged in runs d and f, respectively. For achieving a constant ion strength run d and f were supplemented with 0.5 mmol and 1 mmol Na_2_SO_4_, respectively. Three times the bottle was evacuated and filled with argon, finally ending in a deaerated state. Subsequently the bottle was charged with argon-saturated water (calculated for the end volume of 5 ml), with 0.5 or 1.0 mL argon-saturated 1M Na_2_S solution, with 0.5 (run a und b), 1.0 (run c) or 2.0 ml (run e) 1M NaOH solution and finally with 60 ml CO and 60 ml acetylene, using for the injections gas-tight syringes. To confirm the authenticity of the products, ^13^CO or D_2_O were used in representative experiments. Variations in the initial pH of the reaction batches were induced through the addition of 1M NaOH or 1M H_2_SO_4_. Reactions were carried out at 105 °C.

After 7 days the reaction mixture was allowed to cool down and was centrifuged at 10,000 rpm for 5 minutes. The pH was measured by a glass electrode and 1 ml of the supernatant was freeze-dried. For analysis by GC-MS, the residue was dissolved in 250 μl anhydrous acetonitrile and derivatized with 250 μl N-tert-butyldimethylsilyl-N-methyltrifluoroacetamide for 30 minutes at 70 °C.

Analysis was performed with GC-MS, using GC-2010 coupled with MS-QP2010 Plus (Shimadzu GmbH, D-Duisburg) with a 30 m × 0.25 mm × 0.25 μm fused silica capillary column (Equity TM5, Supelco, Bellefonte, PA, USA) and AOC-20i auto injector. Temperature program and settings:

Program 1: 0–6 min at 60 °C; 6–25 min at 60–280 °C, 10 °C/min; 25–28 min at 280 °C; injector temperature: 260 °C; detector temperature: 260 °C; column flow rate: 1 mL/min; scan interval: 0.5 sec; injection volume 0.2 μl.

Program 2 (used for analysis of C9 acids): 0–6 min at 90 °C; 6–25 min at 90–280 °C, 10 °C/min; 25–28 min at 280 °C;

Otherwise identical to program 1; injection volume 1 μl.

Peak assignment was achieved by comparison of retention times and mass spectra of purchased reference compounds, synthesized products and data from NIST spectra library; for details see Footnotes of Supplemental Table S1.

Quantification was performed by external calibration using known concentrations of commercially available reference compounds (for details see Footnotes of Supplemental Table S1).

All chemicals were purchased from Sigma Aldrich GmbH (D-Steinheim) in the highest purity available. Acetylene was purchased from Linde AG (D-Pullach). Carbon monoxide, Argon 4.6 from Westfalen AG (D-Münster) and ^13^CO from Cambridge Isotopes Laboratories Inc. (USA-MA-Tewksbury).

Heptatrienoic acid was not commercially available and was synthesized by reacting 2 mmol trans-glutaconic acid with 2.2 mmol acrolein in tetrahydrofuran (THF) in the presence of 8 mmol 4-dimethylaminopyridine (DMAP) at 70 °C for 24 h. The product was isolated from the reaction mixture and confirmed by NMR-spectroscopy (AV500 Bruker, Rheinstetten) and GC-MS.

## Additional Information

**How to cite this article**: Scheidler, C. *et al*. Unsaturated C_3,5,7,9_-Monocarboxylic Acids by Aqueous, One-Pot Carbon Fixation: Possible Relevance for the Origin of Life. *Sci. Rep*. **6**, 27595; doi: 10.1038/srep27595 (2016).

## Supplementary Material

Supplementary Information

## Figures and Tables

**Table 1:** Monocarboxylic acid products of the NiS-catalyzed reaction of acetylene with carbon monoxide.

run	a	b	c	d	e	f
mmol NiS	1	1	0.5	0.5	0	0
mmol Ni(OH)_2_ (α or β)	0	0	0.5(α)	0.5(β)	1(α)	1(β)
end-pH	8.8	6.7	8.3	8.9	8.0	9.8
**C**_**3**_**-acids (μM)**						
C_2_H_3_-COOH	3884	5822	3318	6675	250	243
C_2_H_5_-COOH	7132	1069	461	7391	510	171
ΣC_3_	11016	6891	3779	14066	760	414
**C**_**5**_**-acids (μM)**						
ΣC_4_H_5_-COOH	466	1597	970	1463	53	0
ΣC_4_H_7_-COOH	7498	2641	1390	7269	11	0
C_4_H_9_-COOH	309	32	11	363	0	0
ΣC_5_	8273	4270	2371	9095	64	0
**C**_**7**_**-acids (μM)**						
ΣC_6_H_5,7_-COOH	63	30	31	61	0	0
ΣC_6_H_9_-COOH	320	148	69	246	0	0
ΣC_6_H_11_-COOH	52	42	6	96	0	0
ΣC_7_	435	220	106	403	0	0
**C**_**9**_**-acids (μM)**						
ΣC_6_H_5_-C_2_H_2,4_-COOH	1.8	4.4	3.1	1.5	0	0
C_8_H_11_-COOH	0.6	0.8	0.1	0	0	0
ΣC_8_H_13_-COOH	4.6	1.3	3.6	5.9	0	0
C_8_H_15_-COOH	3.9	0.4	0.4	3.7	0	0
ΣC_9_	10.9	6.9	7.2	11.1	0	0
**ΣC**_**3**_**–C**_**9**_	**19735**	**11388**	**6263**	**23575**	**824**	**414**

Reactions were carried out in 125 ml serum bottles with 5 ml aqueous liquid phase for 7 days at 105 °C; Products were identified by GC-MS as *tert*-butyldimethylsilyl derivatives.
