# Supplementary material for: Unsaturated C3,5,7,9-Monocarboxylic Acids by Aqueous, One-Pot Carbon Fixation: Possible Relevance for the Origin of Life
Source: Sci Rep. 2016 Jun 10;6:27595. doi: 10.1038/srep27595 (PMC4901337; doi:10.1038/srep27595)
Supplement: Supplementary Information [file srep27595-s1.pdf]

Unsaturated $C_{3,5,7,9}$-Monocarboxylic Acids by Aqueous, One-Pot Carbon Fixation: Possible Relevance for the Origin of Life

Christopher Scheidler*, Jessica Sobotta*, Wolfgang Eisenreich, Günter Wächtershäuser, Claudia Huber

Supplementary Table S1: Monocarboxylic acid products of the nickel-catalyzed reaction of acetylene with carbon monoxide

Reactions were carried out in 125 ml serum bottles with 5 ml aqueous liquid phase containing 60ml CO and 60ml acetylene for 7 days at 105°C; Products were identified by GC-MS as *tert*-butyldimethylsilyl derivatives;

Acids are classified as follows: yellow: classes of monocarboxylic acids with identical chemical formulae; no colour: individual monocarboxylic acids within said classes; salmon: classes of monocarboxylic acids with identical numbers of C-atoms; red: total sum of identified monocarboxylic acids; blue: hydrogenation yields in % for successive hydrogenation steps, calculated according to the indicated formula (numerals in the formula identify the table rows where the yields for the computation are found).

	run	a	b	c	d	e	f	identification/	ordered characteristic fragment masses			
	mmol NiS	1	1	0.5	0.5	0	0	quantification	(silane fragments 73, 75, 115, 117			
	mmol $Ni(OH)_2$ (α or β)	0	0	0.5(α)	0.5(β)	1(α)	1(β)	methods	etc. not shown)			
	end-pH	8.8	6.7	8.3	8.9	8.0	9.8	Notes	mass 1	mass 2	mass 3	mass 4
	**C_3-acids (μM)**											
1	C_2H_3-COOH	3884	5822	3318	6675	250	243	*, ‡, §, ☆	$129^{3\wedge}$	$55^{3\wedge}$	$85^{3\wedge}$	
2	C_2H_5-COOH	7132	1069	461	7391	510	171	*, ‡, §, ☆	$131^{1\bullet 5\wedge}$			
3	ΣC_3	11016	6891	3779	14066	760	414					
4	2*100/3	65	16	12	53	67	41	%1st hydrogenation				
	**C_5-acids (μM)**											
5	$trCH_2$=CH-CH=CH-COOH	369	1211	742	1212	53	0	*, ‡, §, ☆	$155^{1\bullet 5\wedge}$	$111^{5\wedge}$	$81^{1\bullet 5\wedge}$	$53^{5\wedge}$
6	cCH_2=CH-CH=CH-COOH	97	386	228	251	0	0	*, ‡, §, ††	$155^{1\bullet 5\wedge}$	$111^{5\wedge}$	$81^{1\bullet 5\wedge}$	$53^{5\wedge}$
7	ΣC_4H_5-COOH	466	1597	970	1463	53	0					
8	CH_2=CH-CH_2-CH_2-COOH	830	346	153	946	11	0	*, ‡, §, ‖, #, ☆	$157^{1\bullet 7\wedge}$			
9	$trCH_3$-CH=CH-CH_2-COOH	3791	1565	839	3207	0	0	*, ‡, §, ‖, #, ☆	$157^{1\bullet 7\wedge}$	$113^{7\wedge}$		
10	cCH_3-CH=CH-CH_2-COOH	2053	535	271	1550	0	0	*, ‡, §, ‖, #, ††	$157^{1\bullet 7\wedge}$	$113^{7\wedge}$		
11	CH_3-CH=CH(CH_3)-COOH	578	60	45	1162	0	0	*, ‡, §, ‖, #, ☆	$157^{1\bullet 7\wedge}$			
12	trC_2H_5-CH=CH-COOH	246	135	82	404	0	0	*, ‡, §, ‖, #, ☆	$157^{1\bullet 7\wedge}$	$113^{7\wedge}$	$83^{1\bullet 7\wedge}$	$55^{7\wedge}$
13	ΣC_4H_7-COOH	7498	2641	1390	7269	11	0					
14	C_4H_9-COOH	309	32	11	363	0	0	*, ‡, §, ‖, #, ☆	$159^{1\bullet 9\wedge}$			
15	ΣC_5	8273	4270	2371	9095	64	0					
16	(13+14)·100/15	94	63	59	84	17	—	%1st hydrogenation				
17	14·100/(13+14)	4	1	1	5	0	—	%2nd hydrogenation				
	**C_7-acids (μM) (Note)**											
18	trtrH(CH=CH)$_3$-COOH	26	5	0	24	0	0	†, §, ¶, #, **	181	77	79	59
19	C_6H_5-COOH	38	25	31	37	0	0	*, ‡, #, ☆	$179^{1\bullet 5\wedge}$	$105^{1\bullet 5\wedge}$	$77^{5\wedge}$	$135^{5\wedge}$
20	$\Sigma C_6H_{5\text{-}7}$-COOH	63	30	31	61	0	0					
21	C_6H_9-COOH (a)	19	4	0	42	0	0	‡, §, #, **	$183^{1\bullet 9\wedge}$			
22	C_6H_9-COOH (b)	29	14	8	32	0	0	‡, §, #, **	$183^{1\bullet 9\wedge}$			
23	C_6H_9-COOH (c)	237	92	41	100	0	0	‡, §, #, **	$183^{1\bullet 9\wedge}$			
24	C_6H_9-COOH (d)	35	38	20	72	0	0	‡, §, #, **	$183^{1\bullet 9\wedge}$			
25	ΣC_6H_9-COOH	320	148	69	246	0	0					
26	C_6H_{11}-COOH (a)	25	25	6	32	0	0	‡, §, #, **	$185^{1\bullet}$			
27	C_6H_{11}-COOH (b)	13	7	0	26	0	0	§, #, **	$185^{1\bullet}$	129		
28	C_6H_{11}-COOH (c)	14	10	0	38	0	0	§, #, **	$185^{11\wedge}$			
29	ΣC_6H_{11}-COOH	52	42	6	96	0	0					
30	ΣC_7	435	220	106	403	0	0					
31	(25+29)·100/30	86	86	71	85	—	—	%1st hydrogenation				
32	29·100/(25+29)	14	22	8	28	—	—	%2nd hydrogenation				
	**C_9-acids (μM) (Note)**											
33	C_6H_5-C_2H_4-COOH	0.6	3.01	1.5	0.5	0	0	*, ‡, #, ☆	$207^{1\bullet 9\wedge}$	$91^{7\wedge}$		
34	C_6H_5-C_2H_2-COOH	1.25	1.4	1.56	1.01	0	0	*, ‡, #, ☆	$205^{1\bullet 7\wedge}$	$131^{1\bullet 7\wedge}$	$103^{7\wedge}$	$161^{7\wedge}$
35	ΣC_6H_5-$C_2H_{2\text{-}4}$-COOH	1.85	4.41	3.06	1.51	0	0					
36	C_8H_{11}-COOH	0.61	0.79	0.09	0	0	0	‡, §, #, **	$209^{1\bullet 11\wedge}$	$91^{7\wedge}$		
37	C_8H_{13}-COOH (a)	3.76	0.33	2.47	5.44	0	0	§, #, **	$211^{13\wedge}$	167	109	183
38	C_8H_{13}-COOH (b)	0.80	0.99	1.09	0.48	0	0	§, #, **	211	167	109	137
39	ΣC_8H_{13}-COOH	4.56	1.32	3.56	5.92	0	0					
40	C_8H_{15}-COOH	3.86	0.39	0.37	3.72	0	0	‡, §, #, **	$213^{1\bullet}$	111		
41	ΣC_9	10.9	6.9	7.5	11.1	0	0					
42	(36+39+40)·100/41	83	36	54	87	—	—	%1st hydrogenation				
43	(39+40)·100/(36+39+40)	93	68	98	100	—	—	%2nd hydrogenation				
44	40·100/(39+40)	46	23	9	39	—	—	%3rd hydrogenation				
45	**ΣC_3-C_9**	**19735**	**11388**	**6263**	**23575**	**824**	**414**					

Note: The saturated monocarboxylic acids C_6H_{13}-COOH and C_8H_{17}-COOH were not detected.

* identified by mass spectrum and retention time of a purchased reference compound; † identified by mass spectrum and retention time of a synthesized compound; ‡ identified by D and/or ^{13}C labeling (n• signifies n ^{13}C-labels; n^ signifies n D-labels); § identified by mass spectrum analysis; ‖ identified by its formation from trans-C_4H_5-COOH; ¶ identified by inferred synthetic relationship to C_6H_5-COOH or C_6H_5-$C_2H_{2\text{-}4}$-COOH # identified by relative retention times and ranges of retention times; ☆ quantified by calibration with authentic compound; ** quantified by calibration with closest saturated n-carboxylic acid; †† quantified by calibration with a regioisomer

Supplementary Table S2: Replicates (b′, b″, b‴) of run b. Single values of each detected $C_{3\text{-}9}$ monocarboxylic acid as well as mean values and standard deviations are shown.

**run**	**b´**	**b´´**	**b**	**b´´´**		mean	std. dev.	std.dev [%]
end-pH	6.9	6.9	6.7	6.5				
**C_3-acids (µM)**								
C_2H_3-COOH	4238	3127	5822	5331		4629	1201	26
C_2H_5-COOH	865	586	1069	833		838	198	24
ΣC_3	5102	3714	6891	6164		5468	1381	25
**C_5-acids (µM)**								
trCH_2=CH-CH=CH-COOH	763	971	1090	1271		1024	213	21
cCH_2=CH-CH=CH-COOH	240	331	386	330		322	60	19
ΣC_4H_5-COOH	1003	1302	1476	1601		1345	259	19
CH_2=CH-CH_2-CH_2-COOH	244	200	346	225		253	64	25
trCH_3-CH=CH-CH_2-COOH	1089	1222	1565	1392		1317	207	16
cCH_3-CH=CH-CH_2-COOH	368	473	535	484		465	70	15
CH_3-CH=CH(CH_3)-COOH	85	108	121	141		114	23	21
trC_2H_5-CH=CH-COOH	43	44	60	51		50	8	16
ΣC_4H_7-COOH	1828	2047	2626	2293		2199	343	16
C_4H_9-COOH	27	22	32	27		27	4	15
ΣC_5	2858	3371	4270	3921		3605	620	17
**C_7-acids (µM) (Note)**								
trtrH$(CH=CH)_3$-COOH	3	4	5	6		5	1	27
C_6H_5-COOH	18	24	25	16		20	4	22
$\Sigma C_6H_{5\text{-}7}$-COOH	21	28	30	22		25	4	17
C_6H_9-COOH (a)	3	6	4	2		4	2	43
C_6H_9-COOH (b)	14	12	14	20		15	3	21
C_6H_9-COOH (c)	60	64	92	91		77	17	22
C_6H_9-COOH (d)	19	20	38	34		28	9	34
ΣC_6H_9-COOH	97	102	148	146		123	28	23
C_6H_{11}-COOH (a)	8	20	25	27		20	8	42
C_6H_{11}-COOH (b)	7	1	7	8		6	3	55
C_6H_{11}-COOH (c)	5	7	10	13		9	4	42
ΣC_6H_{11}-COOH	16	28	42	48		34	15	43
ΣC_7	134	158	220	217		182	43	24
**C_9-acids (µM) (Note)**								
C_6H_5-C_2H_4-COOH	3.0	3.0	3.0	4.8		3.5	0.9	26
C_6H_5-C_2H_2-COOH	1.5	1.5	1.4	0.6		1.3	0.4	35
ΣC_6H_5-$C_2H_{2\text{-}4}$-COOH	4.6	4.5	4.4	5.4		4.7	0.5	10
C_8H_{11}-COOH	0.5	0.4	0.8	1.3		0.7	0.4	55
C_8H_{13}-COOH (a)	0.2	0.4	0.3	0.4		0.3	0.1	27
C_8H_{13}-COOH (b)	0.6	0.4	1.0	0.8		0.7	0.3	38
ΣC_8H_{13}-COOH	0.6	0.8	1.3	0.5		0.8	0.4	46
C_8H_{15}-COOH	0.2	0.3	0.4	0.3		0.3	0.1	22
ΣC_9	5.8	4.4	6.9	7.8		6.2	1.5	24
**$\Sigma C_{3\text{-}9}$**	**8100**	**7247**	**11388**	**10321**		**9261**	**1917**	**21**

Note: The saturated monocarboxylic acids C_6H_{13}-COOH and C_8H_{17}-COOH were not detected
Identification and quantification was performed as stated in Supplementary Table S1.

Supplementary Table S3: Mol% conversion of acetylene and mol% consumption of CO for production of monocarboxylic acids according to run d:

—The stoichiometric conversion of C_2H_2 into C_n-carboxylic acids (n = 3, 5, 7, 9) is determined as follows: The individual concentrations (µM) are multiplied by $5 \cdot 10^{-3}$ ml (to obtain µmol/5 ml) and by the factor 0.5(n-1) that accounts for the number of C_2H_2 molecules that entered the carbon chain.

—The stoichiometric consumption of CO for formation of C_n-carboxylic acids (n = 3, 5, 7, 9) is determined as follows: The individual concentrations (µM) are multiplied by $5 \cdot 10^{-3}$ ml and by the factor (1 + x), whereby x represents the number of hydrogenated double bonds.

			**mol% conversion**		**mol% consumption**		
	[µM]	µmol/5 ml	factor	acetylene	factor	CO	product name
C_2H_3-COOH	6675	33.38	1	1.38	1	1.38	Acrylic acid
C_2H_5-COOH	7391	36.96	1	1.53	2	3.05	Propionic acid
ΣC_3 acids	14066	70.33		2.91		4.43	
ΣC_4H_5-COOH	1463	7.315	2	0.60	1	0.3	Pentadienoic acid
ΣC_4H_7-COOH	7269	36.34	2	3.0	2	3.0	Pentenoic acid
C_4H_9-COOH	363	1.82	2	0.15	3	0.23	Pentanoic acid
ΣC_5 acids	9094	45.47		3.77		3.46	
trtrH(CH=CH)$_3$-COOH	24	0.12	3	0.015	1	0.005	Heptatrienoic acid
C_6H_5-COOH	37	0.19	3	0.024	1	0.008	Benzoic acid
ΣC_6H_9-COOH	246	1.23	3	0.15	2	0.102	Heptadienoic acid
ΣC_6H_{11}-COOH	96	0.48	3	0.06	3	0.06	Heptenoic acid
ΣC_7 acids	403	2.02		0.249		0.175	
C_6H_5-C_2H_4-COOH	0.5	0.0025	4	0.0004	2	0.0002	Hydrocinnamic acid
C_6H_5-C_2H_2-COOH	1.01	0.0051	4	0.0008	1	0.0002	Cinnamic acid
C_8H_{11}-COOH	0	0		0		0	Nonatrienoic acid
ΣC_8H_{13}-COOH	5.9	0.0295	4	0.0049	3	0.0037	Nonadienoic acid
C_8H_{15}-COOH	3.7	0.0185	4	0.0031	4	0.0031	Nonenoic acid
ΣC_9 acids	11.1	0.0555		0.0092		0.0072	
**ΣC_3-C_9 acids**	**23574**	**118**		**6.94**		**8.07**	

Supplementary Figure S4:
GC-MS chromatogram of run d in comparison to control runs without acetylene and/or without CO are shown; segments for C_3, C_5, C_7 and C_9 acids are shown separately.

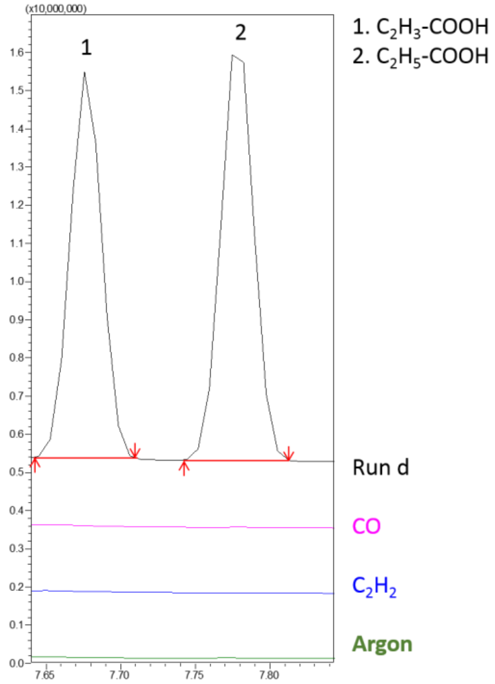

Figure S4a: GC-MS chromatogram of C_3 monocarboxylic acids of run d in comparison to three control runs.
Control runs were carried out identical to run d containing only 120 ml CO (pink) or 120 ml acetylene (blue) or only 120 ml argon (green). After 7 days at 105°C products were identified by GC-MS as *tert*-butyldimethylsilyl derivatives with GC-MS program 1 (0-6 min at 60 °C; 6-25 min at 60-280 °C, 10 °C/min; 25-28 min at 280 °C; injector temperature: 260 °C) .

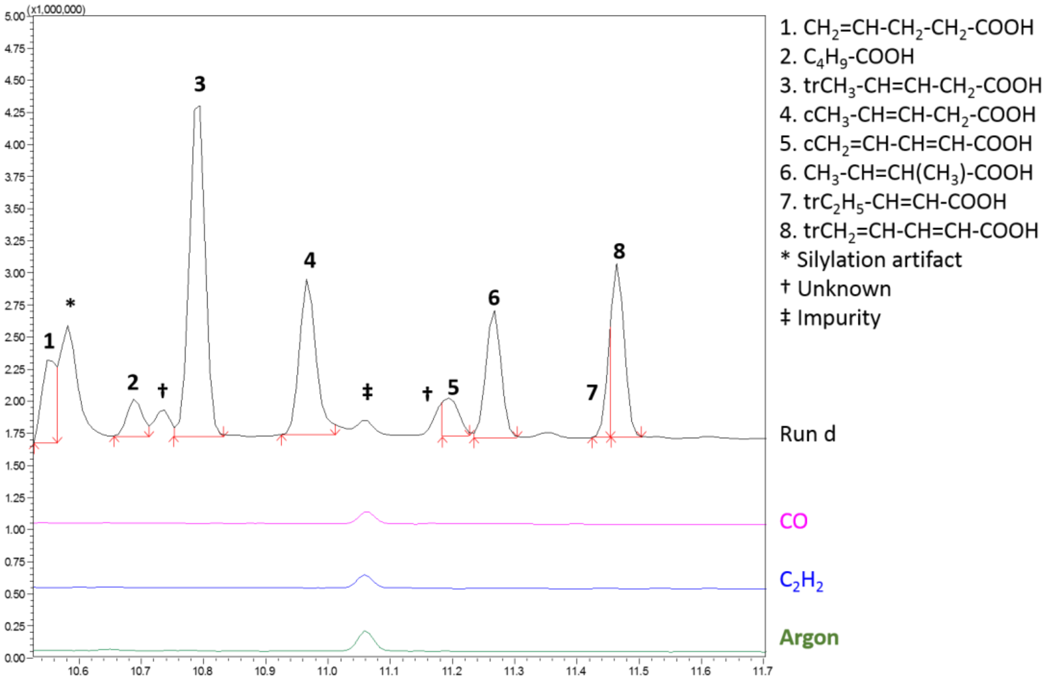

Figure S4 b: GC-MS chromatogram of C_5 monocarboxylic acids of run d in comparison to three control runs.
Control runs were carried out identical to run d containing only 120 ml CO (pink) or 120 ml acetylene (blue) or only 120 ml argon (green). After 7 days at 105°C products were identified by GC-MS as *tert*-butyldimethylsilyl derivatives with GC-MS program 1 (0-6 min at 60 °C; 6-25 min at 60-280 °C, 10 °C/min; 25-28 min at 280 °C; injector temperature: 260 °C) .

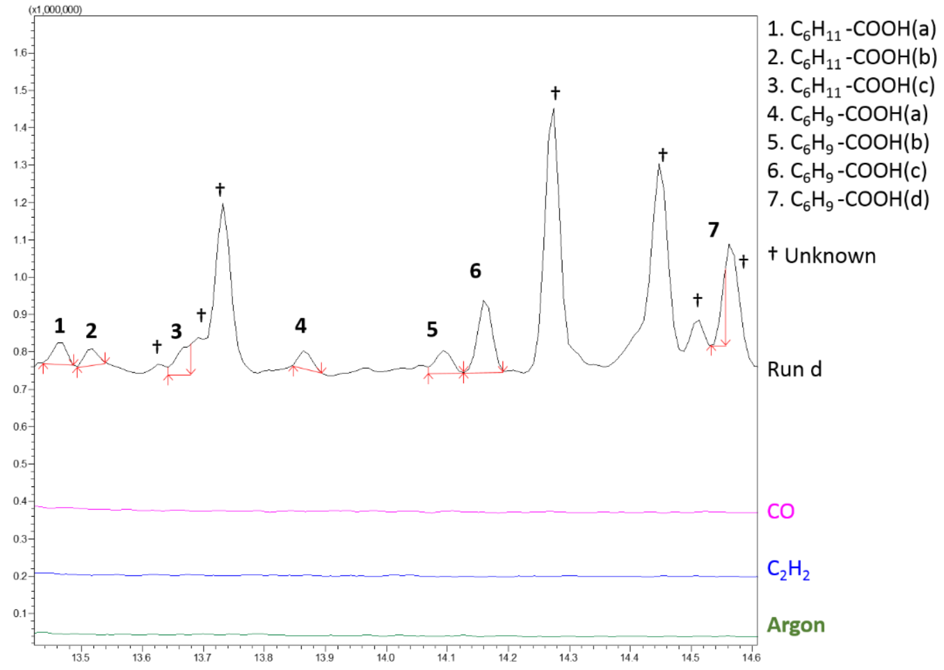

Figure S4 c: GC-MS chromatogram of C_7 monocarboxylic acids of run d in comparison to three control runs.
Control runs were carried out identical to run d containing only 120 ml CO (pink) or 120 ml acetylene (blue) or only 120 ml argon (green). After 7 days at 105°C products were identified by GC-MS as *tert*-butyldimethylsilyl derivatives with GC-MS program 1 (0-6 min at 60 °C; 6-25 min at 60-280 °C, 10 °C/min; 25-28 min at 280 °C; injector temperature: 260 °C).

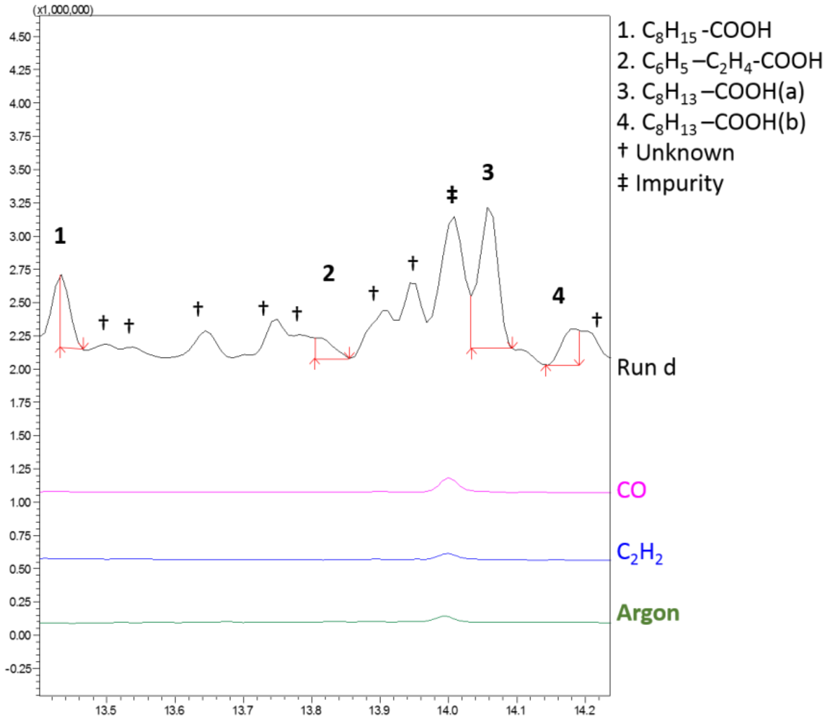

Figure S4 d: GC-MS chromatogram of C_9 monocarboxylic acids of run d in comparison to three control runs.
Control runs were carried out identical to run d containing only 120 ml CO (pink) or 120 ml acetylene (blue) or only 120 ml argon (green). After 7 days at 105°C products were identified by GC-MS as *tert*-butyldimethylsilyl derivatives with GC-MS program 2 (0-6 min at 90 ºC; 6-25 min at 90-280 ºC, 10 ºC/min; 25-28 min at 280 ºC).
